# Breakthroughs and Opportunities of Biopolymer Coatings: A Bibliometric Analysis on Their Future Prospects for the Sustainable Food Packaging Industry

**DOI:** 10.1155/tswj/5337593

**Published:** 2025-11-12

**Authors:** Huda Mohamad Elmatsani, Wahyu Bahari Setianto, Nasruddin Nasruddin, Heryoki Yohanes, Nur Sri Wahyuni, Rudiyono Rudiyono, Puji Astuti, Eko Bhakti Susetyo, Mochammad Jusuf Djafar, Astuti Astuti, Arief Arianto, Lanjar Lanjar

**Affiliations:** ^1^Research Center for Agroindustry, National Research and Innovation Agency (BRIN), KST Bacharuddin Jusuf Habibie, Tangerang Selatan, Banten, Indonesia; ^2^Research Center for Polymer Technology, National Research and Innovation Agency (BRIN), KST Bacharuddin Jusuf Habibie, Tangerang Selatan, Banten, Indonesia; ^3^Directorate of Laboratory Management, Research Facilities, and Science and Technology Parks (DPLFRKST), National Research and Innovation Agency (BRIN), KST Bacharuddin Jusuf Habibie, Tangerang Selatan, Banten, Indonesia

**Keywords:** bibliometric analysis, biodegradable packaging, biopolymer coating, environmental sustainability, food preservation, global research trends

## Abstract

Plastic packaging poses significant environmental challenges due to its resistance to natural degradation. In response, there has been a growing body of research dedicated to developing sustainable packaging alternatives, particularly those based on biodegradable materials. Biopolymer-based packaging is widely recognized for its enhanced environmental compatibility, owing to its capacity for natural decomposition and reduced ecological footprint. This study presents a comprehensive bibliometric analysis of recent advancements in biopolymer coatings for food packaging, with a focus on their potential to enhance food safety, quality, and shelf life. Analysis of publications from 2015 to 2024 indicates a substantial and consistent rise in scholarly output related to biopolymer-based packaging solutions, driven by global efforts to mitigate plastic waste and its associated environmental impacts. The findings suggest that the future trajectory of biodegradable packaging research should emphasize the development of materials that simultaneously support human health and environmental sustainability. Bibliometric mapping identified polylactic acid (PLA), polyhydroxyalkanoates (PHAs), chitosan, and cellulose-based polymers as key areas of innovation, highlighting their increasing prominence and interdisciplinary research significance. These materials are notable for their biodegradability, and many also exhibit intrinsic functional properties such as antimicrobial and antioxidant activities—attributes that are highly desirable in food packaging applications. Biopolymer coatings, in particular, have demonstrated excellent barrier properties, including controlled permeability to moisture and oxygen, which are critical for maintaining food integrity. Such coatings contribute to extended shelf life by minimizing dehydration and oxidative damage while concurrently offering protection against microbial contamination. Despite the promising functional characteristics of these materials, several challenges continue to hinder their widespread adoption. Key barriers include high production costs, the limited availability of industrial composting infrastructure, and the general lack of consumer awareness regarding appropriate disposal practices. Addressing these challenges will require coordinated efforts across research, industry, and policy domains. To enable scalable implementation, future innovation must achieve critical advancements in materials science, biopolymer production efficiency, and regulatory alignment. A comprehensive, multidisciplinary approach will be essential to realize the full potential of biopolymer coatings as sustainable alternatives to conventional plastic packaging.

## 1. Introduction

Concerns about environmental pollution have increased significantly. The need to address issues associated with conventional plastic packaging has become increasingly urgent [1]. These factors have driven significant transformations in the global food packaging industry. Conventional food coatings made from plastics like polyethylene (PE), polypropylene (PP), and PE terephthalate (PET) are effective in preserving food quality. However, these materials pose significant environmental and health risks. Their lack of biodegradability and potential for chemical leaching are major concerns [2].

Plastics contain chemicals such as Bisphenol A (BPA) and phthalates, which are linked to hormone disruption and other health issues [3]. Additionally, substances like BPA, phthalates, and styrene are associated with increased cancer risk [4]. The risk of contamination increases when plastic coatings are exposed to heat. Heat accelerates the leaching of these carcinogenic substances into packaged foods [5]. While these plastic coatings are effective for food preservation, their health and environmental hazards call for safer alternatives [6].

The growing environmental and health concerns surrounding conventional plastics have significantly intensified the search for sustainable alternatives in food packaging. Biopolymer coatings, sourced from renewable polysaccharides, proteins, and lipids, offer a biodegradable, functional alternative to petroleum-based materials [7, 8]. These coatings help reduce environmental waste while extending food shelf life by acting as effective barriers to moisture, oxygen, and microbial contamination [9, 10]. The inherent biodegradability of biopolymer coatings ensures that they break down naturally, mitigating the problem of micro- and nanoplastic pollution, while their functional properties help preserve food quality and safety [7, 11]. As the demand for more sustainable alternatives to plastic packaging grows, these biopolymer coatings have become ideal candidates for reducing the environmental footprint of packaging waste while maintaining food quality. Recent advancements in technology have also facilitated the integration of smart packaging features into biopolymer coatings, enabling them to transmit real-time data to consumers, monitor food freshness, and further minimize the environmental impact of packaging waste [11–13]. These innovations position biopolymer coatings as key technologies in the transition to sustainable, efficient, and environmentally friendly food packaging.

Advancements in biotechnology and materials science have enhanced the mechanical properties and biodegradability of these polymers, making them more suitable for large-scale commercial applications [14–16]. Furthermore, the integration of natural fibers and bio-based additives into biodegradable polymers has further enhanced their performance and sustainability [2]. The inclusion of cellulose- and starch-based materials is particularly effective in enhancing film strength and biodegradability, making them a more sustainable alternative to conventional plastics [17–19].

With the global push for sustainable alternatives, regulatory frameworks are increasingly favoring eco-friendly packaging solutions. Continued research and technological advancements for biopolymer coatings will be critical in accelerating commercialization and scalability [20, 21], especially active packaging systems that incorporate antimicrobial agents and antioxidants to extend shelf life and maintain food quality [22, 23]. These innovations also support the dual goals of reducing plastic waste, promoting a circular economy by utilizing agricultural byproducts, and reducing reliance on fossil fuels [7, 24, 25].

However, despite these advancements, several challenges hinder the widespread adoption of biopolymer coatings. These challenges include higher production costs compared to conventional plastics, limited industrial composting infrastructure, and the need for consumer education on proper disposal methods [26]. Furthermore, the environmental benefits of biodegradable plastics are closely tied to the conditions under which they are disposed of, highlighting the importance of developing efficient waste management systems [27].

Numerous studies have explored the mechanical, thermal, and biodegradability properties of biopolymer coatings; however, the existing literature remains largely experimental and fragmented. A notable gap exists in systematically linking these technological advancements to real-world industry adoption, particularly in sustainable food packaging. While bibliometric analyses in polymer science and biopolymers have been conducted [28, 29], they have largely focused on general polymers, neglecting the unique challenges and characteristics of biopolymer coatings, especially in food packaging applications. This article stands out by applying bibliometric analysis specifically to this underexplored domain. Unlike traditional reviews that summarize existing research, this study employs a systematic, data-driven approach to map the evolution of research in biopolymer coatings for food packaging. It identifies key contributors, uncovers emerging research areas, and highlights critical knowledge gaps. Through this quantitative framework, the study presents opportunities for innovation within the food packaging sector, advancing the development of more sustainable solutions [30, 31].

This review addresses a significant gap in the literature by offering a comprehensive bibliometric analysis focused on biopolymer coatings for food packaging. Although there is a growing body of research on biopolymers in packaging, much of this work is experimental and disconnected from real-world applications. By zeroing in on biopolymer coatings, this study provides an in-depth overview of current research trends, identifies areas ripe for further exploration, and presents key takeaways for researchers, policymakers, and industry stakeholders. Readers will gain a holistic understanding of key advancements, ongoing challenges, and emerging opportunities, equipping them to make informed decisions that can shape future efforts in sustainable food packaging.

With a specific focus on biopolymer coatings, this review brings fresh perspectives to the development of sustainable materials, emphasizing the integration of bioactive and eco-friendly components that enhance both functionality and environmental sustainability [32, 33]. It not only bridges the gap between academic research and industry needs but also uncovers critical, underexplored opportunities for innovation. The findings of this study are particularly valuable for those seeking to advance sustainable food packaging technologies, address emerging challenges, and guide future developments in biopolymer coating technologies [34, 35]. Ultimately, this review lays the groundwork for continued progress, supporting the integration of biopolymer coatings into food packaging systems and fostering the development of environmentally sustainable and functionally superior solutions.

The contributions of this study are substantial and multidimensional. First, it offers a thorough summary of the current state of research on biopolymer coatings, helping researchers and industry professionals to understand the major trends and gaps in the literature. This research also offers critical insights into technological advancements that could enhance the commercial viability of biopolymer coatings, making them more competitive with traditional plastics. By identifying key challenges and proposing solutions for scaling and integrating biopolymer coatings into the food packaging industry, this study addresses significant barriers to their adoption.

## 2. Method

### 2.1. Research Design

This study employed a bibliometric analysis to systematically examine the research landscape of biopolymer coatings for food packaging. The analysis focuses on key research trends, technological advancements, barriers to adoption and scalability, and the environmental and economic impacts of biopolymer coatings. The data for this study were collected from the Scopus database, which is known for its extensive coverage of peer-reviewed literature across multiple disciplines. Scopus was chosen due to its high-quality indexing and robust analytical tools, ensuring the reliability and accuracy of the bibliometric analysis.

Two primary analytical approaches were used: performance analysis and scientific mapping. Performance analysis assessed research productivity and impact using metrics such as publication count, citation metrics, and the identification of leading contributors in the field. Scientific mapping visualized the relationships among research elements such as keyword co-occurrences, cocitations, and author collaborations. Tools like VOSviewer and Bibliometrix were employed to create network maps and perform statistical analyses. Together, these methods provided a detailed examination of the research landscape, highlighting trends, gaps, and opportunities for future research in biopolymer coatings for food packaging.

### 2.2. Data Collection

#### 2.2.1. Search Strategy

**Figure 1:**
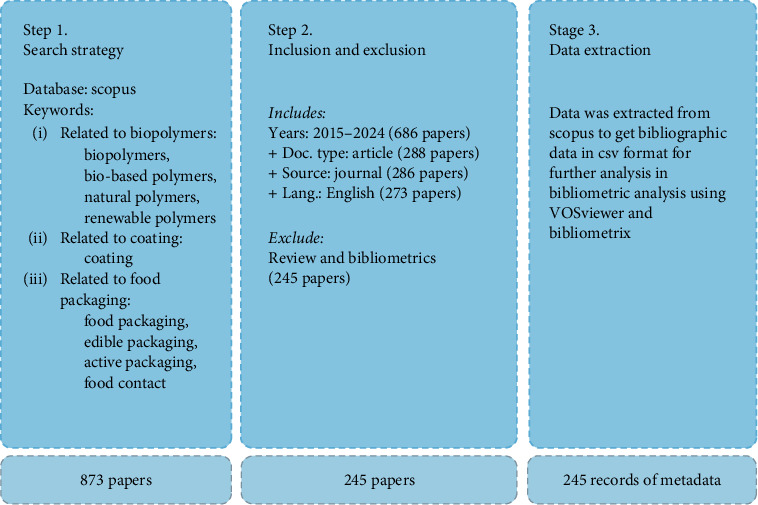
Data collection procedure involving keyword-based Scopus searches, inclusion/exclusion criteria, and metadata extraction for 245 papers (2015–2024), analyzed using VOSviewer and Bibliometrix to explore biopolymer coating trends.

The data collection process began by identifying relevant publications through a targeted search on Scopus. This search utilized key terms related to biopolymer coatings and food packaging to focus on research within this specific area, as shown in Figure 1. To capture the latest developments and emerging trends, the search was limited to peer-reviewed articles and conference papers published between 2015 and 2024. This approach ensured the inclusion of the most current advancements in the field.

#### 2.2.2. Inclusion and Exclusion

To ensure the relevance and quality of the collected data, strict inclusion and exclusion criteria were applied. The study focused exclusively on publications that specifically addressed biopolymer coatings within the context of food packaging. Studies that did not relate to this application or lacked substantial analysis were excluded from the dataset. Additionally, review papers were excluded to maintain a focus on original research. This approach allowed the study to concentrate on articles that present new data, findings, and technological advancements, thereby capturing the evolution of scientific contributions and the innovative research driving the field forward.

The following query was applied in the Scopus database search to refine the selection process:

(TITLE-ABS-KEY (Biopolymer OR “Bio-based polymers” OR “Natural polymers” OR “Renewable polymers”) AND TITLE-ABS-KEY (“coating”) AND TITLE-ABS-KEY (“food packaging” OR “edible packaging” OR “active packaging” OR “food contact”)) AND PUBYEAR >2014 AND PUBYEAR <2025 AND NOT TITLE-ABS-KEY (“bibliometric” OR “review”) AND (LIMIT-TO (SRCTYPE, “j”)) AND (LIMIT-TO (DOCTYPE, “ar”)) AND (LIMIT-TO (LANGUAGE, “English”))

The Scopus dataset included essential metadata, such as titles, authors, publication years, journals, keywords, abstracts, and citation data, ensuring a robust foundation for the analysis.

#### 2.2.3. Data Extraction

Once relevant studies were identified, detailed metadata for each publication were extracted. This metadata included information such as titles, authors, publication years, journals, keywords, abstracts, citation data, and references. This comprehensive dataset forms the foundation for the subsequent bibliometric analysis, enabling a thorough exploration of research trends, technological innovations, and the wider impact of biopolymer coatings in the food packaging industry. The extracted data were structured to facilitate both performance analysis and scientific mapping, ensuring that the analysis provides a clear and detailed understanding of the research landscape.

### 2.3. Analysis and In-Depth Review

**Figure 2:**
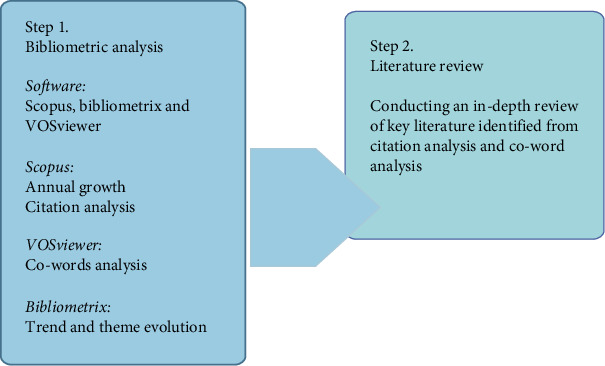
Analysis steps, combining bibliometric analysis using Scopus, VOSviewer, and Bibliometrix, followed by an in-depth literature review to explore key trends and themes.

The analysis was conducted using performance analysis and scientific mapping techniques. Performance analysis evaluated research output and impact, while scientific mapping visualized the relationships between research elements, as illustrated in Figure 2. The collected data were further analyzed to identify key trends, influential studies, and emerging research areas.

To ensure continuity between the data collection and analysis phases, the extracted metadata were structured to facilitate both performance analysis and scientific mapping. The use of tools like VOSviewer enabled the visualization of research clusters and key areas of collaboration, offering a more detailed understanding of the development of biopolymer coatings for food packaging. The analysis was driven by the study's objectives of identifying research trends, assessing technological advancements, and uncovering gaps in the literature that could inform future research directions.

To deepen the analysis, a focused literature review was conducted, selecting key studies based on their citation count and content relevance. This ensured that the review concentrated on significant and highly pertinent research, particularly on technological advancements and the integration of nanomaterials and natural fibers in biopolymer coatings. By critically evaluating these studies, the review provided new insights and theoretical developments, reinforcing the bibliometric findings and highlighting existing gaps and areas for future research. The combination of these methodologies provided a comprehensive analysis of the research landscape, allowing for the identification of trends and the assessment of the potential of biopolymer coatings in food packaging.

### 2.4. Interpreting the Findings and Results

This study offers academically rigorous insights into biopolymer coatings for the food packaging industry, highlighting trends like material property advancements, natural fiber integration, and active packaging systems. Technological advancements, particularly in improving mechanical strength, biodegradability, and cost-effectiveness, were contextualized for commercial applications. The study critically analyzed challenges like high production costs and limited composting infrastructure, proposing strategies to overcome them. Environmental and economic impacts were evaluated, highlighting biopolymer coatings' potential role in the circular economy, with recommendations for scaling and integrating these solutions to support sustainable packaging.

## 3. Result and Discussion

### 3.1. Annual Scientific Publication

**Figure 3:**
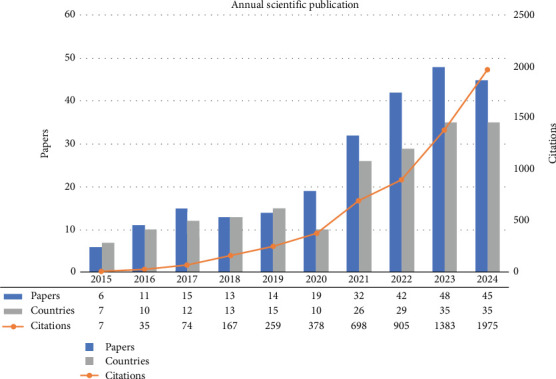
The increase in publications demonstrates the high interest of researchers in developing biopolymers as environmentally friendly solutions in food packaging.

Figure 3 illustrates a significant increase in annual scientific publications related to biopolymer coatings for food packaging from 2015 to 2024. This upward trend signals a growing interest and active research in developing sustainable alternatives to conventional packaging. The dataset, comprising 245 documents, reveals a notable rise in publications, particularly after 2020, with an annual growth rate of 24.78%. The graph also shows an exponential increase in citations, emphasizing the growing attention researchers are giving to the topic of biopolymer coatings for food packaging. From just six papers in 2015, the number of publications has steadily expanded. The growing research activity in biopolymer coatings reflects global efforts to combat plastic pollution, as seen in international regulations like the European Union's single-use plastic ban and the UN's Sustainable Development Goals, to mitigate plastic pollution in marine environments [28, 29]. These efforts aim to reduce plastic pollution in marine environments [28, 29], which has severe consequences for oceans. Marine organisms ingest microplastics, leading to physical harm, chemical contamination [36], habitat disruption, and biodiversity loss [37]. Additionally, human health is at risk as microplastics enter the food chain through seafood consumption [38].

Given these environmental concerns, the research on biopolymer coatings for food packaging is increasingly important. Sustainable alternatives to plastics are critical to reducing environmental impact and promoting ecosystem health [39]. This increase in research is reflected in a rising number of citations, indicating that these studies are having a notable influence and providing valuable insights to the field. Citations grew from 7 in 2015 to 1975 in 2024, highlighting the growing impact of this research. Tracking publication and citation trends will be crucial for steering future research directions and sustaining ongoing advancements [40].

### 3.2. Publication by Journal and Country

#### 3.2.1. The Most Prominent Journals

Table 1 highlights leading journals in biopolymer coatings for food packaging, underscoring their significant contributions to advancing sustainable food preservation. The *International Journal of Biological Macromolecules* is at the forefront of research on chitosan (CH), a biopolymer known for its antimicrobial and biodegradable properties, which enhances food packaging. This journal has played a crucial role in developing coatings that improve food quality and shelf life [41]. Similarly, *Progress in Organic Coatings* has focused on enhancing CH coatings by incorporating photocatalysts like ZnO@SnOx nanoparticles, expanding their functionality, and improving antimicrobial properties [47].

*Polymers* journal has made notable advancements in the use of natural-based polymers, particularly cellulose, by investigating the conversion of asparagus stalk cellulose into carboxymethyl cellulose (CMC), a promising material for food packaging [42]. Similarly, *Food Packaging and Shelf Life* has explored bio-based coatings such as hydroxypropyl methylcellulose, guar gum, and potassium sorbate, which help preserve fruit quality and inhibit fungal growth, offering practical solutions for extending shelf life [45]. While both journals focus on bio-based materials, *Polymers* journal emphasizes their structural potential, whereas *Food Packaging and Shelf Life* highlights their functional applications.

*Food Chemistry* has made notable contributions to edible coatings, especially those based on hydroxypropyl methylcellulose and beeswax, which effectively control ripening, preserve fruit quality, and extend shelf life, particularly for mangoes [44]. This journal distinguishes itself by combining food preservation with biodegradable, edible coatings, complementing the structural and functional approaches found in *Polymers* journal and *Food Packaging and Shelf Life*.

*Coating* and *Progress in Organic Coatings* have both made valuable contributions to sustainable packaging research. *Coating* has examined xylan derivatives and CH coatings, which enhance the barrier and antimicrobial properties of paper. Meanwhile, *Progress in Organic Coatings* has advanced photocatalyst-loaded CH coatings, further improving antimicrobial functionality [46, 47]. Additionally, *Food Hydrocolloids* introduced a simple coating method using CH, glutinous rice starch, and polylactic acid (PLA) to create paper for food packaging that is water-resistant, biodegradable, and recyclable. This method complements the findings in the *Journal of Applied Polymer Science*, which demonstrated that biopolymer-coated paper offers improved transparency and enhanced barrier properties, particularly against water and limonene vapors [43]. Together, these studies highlight the effectiveness of biopolymer coatings in enhancing both the physical properties and barrier performance of paper packaging.

#### 3.2.2. The Most Productive Countries

Table 2 presents the top 10 countries in biopolymer coating research, ranked by the number of publications, offering valuable insights into global research trends in this rapidly growing field. Brazil leads the list with 29 papers, highlighting its strong commitment to biopolymer coating research. This leadership is further underscored by the 1265 citations, suggesting that Brazilian research is not only prolific but also influential. The most cited Brazilian paper by Espitia et al. highlights the country's emphasis on the practical applications of biopolymers, particularly in food packaging. The study explores the use of nanoemulsions to enhance biopolymer coatings, enabling the incorporation of bioactive compounds such as essential oils and vitamins into food packaging materials [51]. This emphasis on real-world applications likely contributes to the high citation rate, indicating that Brazilian researchers are effectively addressing relevant industrial and environmental challenges.

China ranked second with 28 papers and 833 citations, demonstrating its significant influence in biopolymer coating research, including nanotechnology applications. A key study by Guo et al., cited 92 times, explored cinnamaldehyde and zinc oxide nanoparticles (ZnONPs) in CMC-based coatings to improve cherry tomato postharvest quality, showcasing advancements in nanotechnology for food packaging [52].

In comparison, countries such as Italy, India, and Spain have also made notable contributions. Italy and India each produced 27 papers, but Italy's higher citation impact, with 985 citations, indicates a greater influence relative to its output. Italy's focus on cellulose nanocrystals (CNCs), exemplified by the work of Mascheroni et al., highlights the country's expertise in materials science and packaging applications [53]. In contrast, countries like Thailand and Iran, with lower publication and citation numbers, indicate emerging interest in biopolymers. Despite this, their research topics—such as multilayer edible coatings using electrostatic methods in Thailand and the use of rosemary essential oil via electrospinning techniques in Iran—underscore the global nature of biopolymer research and the diverse approaches being explored in the field.

### 3.3. Citation Analysis

Table 3 highlights the top 10 influential papers in the field of biopolymer coatings for food packaging, emphasizing key advancements in developing sustainable and functional packaging solutions. These studies focus on a variety of biopolymers, such as CH, CNCs, and alginates, and the incorporation of additives like essential oils and nanomaterials to enhance the performance of packaging materials.

CH-based coatings are widely recognized for their biodegradability, antimicrobial properties, and film-forming capabilities. These coatings have been shown to effectively reduce weight loss and lipid oxidation in beef and provide antibacterial protection for strawberries [61, 62]. The incorporation of essential oils or nanomaterials into CH-based films further improves their functionality, as demonstrated by Homez-Jara et al., who optimized CH coatings by adjusting drying temperatures and concentrations to enhance their mechanical properties [41].

In addition to CH, CNCs have emerged as a key material for food packaging. Mascheroni et al. demonstrated the potential of CNC coatings, produced through ammonium persulfate treatment, to improve oxygen barrier properties and transparency [53]. CNCs, derived from natural cellulose fibers, offer superior mechanical strength, higher crystallinity, and strong hydrogen bonding, contributing to improved product integrity and shelf life, making them a promising biopolymer material for sustainable packaging.

The incorporation of essential oils, such as thyme essential oil, into CH coatings has further enhanced the antimicrobial properties of biopolymer films. Al-Moghazy et al. showed that CH films with thyme essential oil encapsulated in liposomes extended the shelf life of Karish cheese by maintaining microbial safety for up to 4 weeks [65]. These studies underscore the dual role of biopolymer coatings in improving both mechanical and antimicrobial properties, offering a promising alternative to traditional food packaging while meeting growing demands for sustainability and food safety.

### 3.4. Keyword Clustering and Research Area Mapping

**Figure 4:**
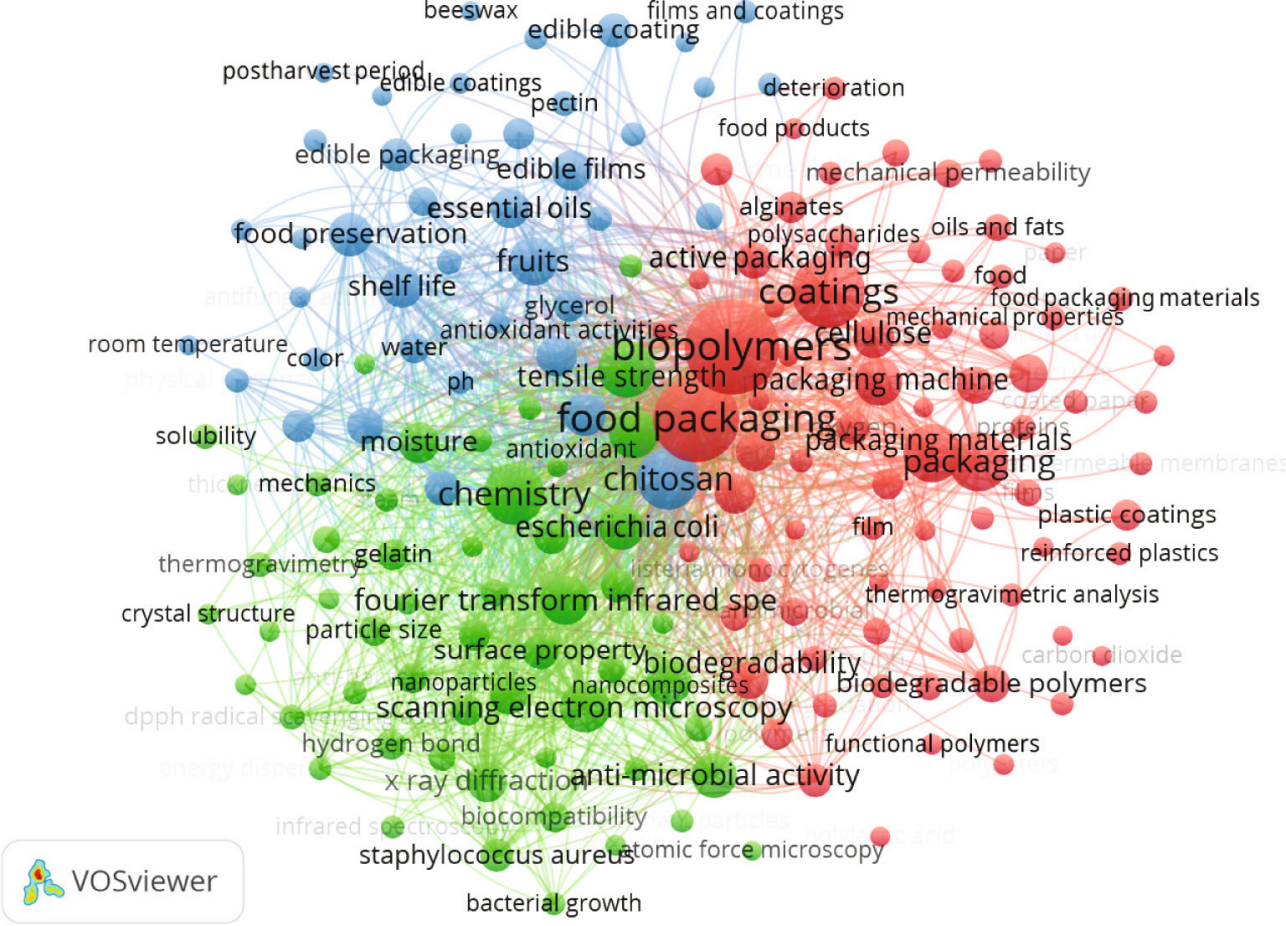
Co-occurrence map of biopolymer coatings for food packaging, highlighting interconnected topics such as biopolymers, food packaging, mechanical properties, and biodegradability, generated using VOSviewer co-word analysis.

The co-occurrence analysis of keywords using VOSviewer (Figure 4) provides a comprehensive examination of the current research landscape in biopolymer coatings for food packaging, identifying key themes and emerging trends within the field. This analysis categorizes 170 keywords into three distinct clusters, each representing different aspects of biopolymer coating research.  Table 4 summarizes the thematic clustering results from the co-occurrence analysis of keywords in publications on biopolymer coatings for food packaging. The clusters—red, green, and blue—represent distinct research foci: (1) biopolymers and sustainability in food packaging, (2) material functionality and characterization techniques, and (3) applications in food preservation, safety, and shelf life enhancement. Each cluster groups key terms that frequently co-occur in the literature, highlighting dominant themes and research priorities within the field.

The red cluster is centered on biopolymer coatings for sustainable food packaging. It highlights key topics such as biopolymers, biodegradable polymers, coatings, food packaging, and active packaging. Research in this cluster focuses on optimizing natural polymer–based materials, such as cellulose, alginate, and polysaccharides, to replace synthetic packaging materials and improve sustainability.

Studies have demonstrated that cellulose derivatives like CMC, synthesized from *Asparagus officinalis* stalks [42], offer enhanced mechanical properties and biodegradability for food packaging. Similarly, alginate modifications, including amidation [66], improve water barrier properties, making them suitable for moisture-sensitive food applications. The incorporation of nanomaterials like ZnONPs and gallic acid (GA) in methylcellulose films has also been shown to enhance UV barrier capacity, mechanical strength, and antimicrobial activity [67]. Moreover, natural extracts such as grape marc and moringa leaf in cellulose-based coatings have proven effective in reducing lipid peroxidation and maintaining food quality during storage [68]. These advances highlight the potential of natural, biodegradable coatings enhanced with bioactive agents and nanoparticles to provide functional, sustainable food packaging solutions while ensuring food safety and extending shelf life.

The green cluster focuses on enhancing the functional properties of biopolymer coatings through the integration of nanotechnology and advanced characterization techniques. Studies have made significant advancements in improving the mechanical strength, thermal stability, and antimicrobial properties of biopolymer films, with key methods like scanning electron microscopy (SEM) and Fourier transform infrared spectroscopy (FTIR) used to assess film characteristics. The incorporation of nanoparticles such as ZnONPs and silver nanoparticles (AgNPs), along with essential oils like thymol, has shown substantial improvements. For instance, ZnONPs in CMC films improved barrier properties and antifungal activity [52], while AgNPs in poly(3-hydroxybutyrate) films enhanced antimicrobial activity against foodborne pathogens like *Salmonella* and *Listeria* [69]. These additions enhance the durability and antimicrobial properties of food packaging materials, making them more effective in maintaining food safety and quality.

Additionally, the incorporation of bioactive agents such as essential oils has proven beneficial for food preservation. Thymol-loaded nanoemulsions in egg white protein–based films demonstrated sustained antibacterial activity against *Staphylococcus aureus* and *Escherichia coli* [70], while essential oils in sago starch and guar gum films showed significant antimicrobial effects [64]. These innovations not only improve the mechanical and antimicrobial properties of biopolymer films but also contribute to sustainable packaging solutions. Overall, the integration of nanoparticles and bioactive agents enhances the functional properties of biopolymer coatings, positioning them as viable, eco-friendly alternatives in food packaging to ensure food safety and extended shelf life.

The blue cluster emphasizes the practical applications of biopolymer coatings, particularly in food packaging and edible coatings. Research in this cluster underscores the significance of shelf life extension, food preservation, and food safety, with key keywords such as CH, fruits, edible films, essential oils, and antioxidants central to the discourse. Studies demonstrate how biopolymer coatings, such as CH–gelatin and pectin–gelatin films, are effective in improving the storage conditions of perishable food products. For example, CH–gelatin-based films were shown to reduce weight loss and lipid oxidation in beef steaks during retail display while enhancing color stability and preserving food quality [61]. Similarly, sodium alginate (NaAlg) and locust bean gum (LBG) coatings effectively preserved the postharvest quality of Valencia oranges and reduced microbial growth by more than 73% [71].

These coatings, often enriched with bioactive agents like essential oils (e.g., lemongrass essential oil [LEO] and eugenol), provide additional protection against microbial contamination and oxidative spoilage. For instance, CH–gelatin films incorporating LEO demonstrated antibacterial activity and extended the shelf life of raspberries during storage [72]. The integration of green AgNPs (G-AgNPs) into cornstarch-based films also showed a significant reduction in weight loss in cherry tomatoes and grapes, emphasizing the effectiveness of nanocomposites in enhancing food safety and shelf life [73]. Furthermore, aloe vera gel (AV) and CH films improved the water solubility, thermal properties, and antioxidant properties, enhancing the preservation of fresh figs [74]. These innovations highlight the role of edible coatings and active packaging in extending the shelf life and quality of food products while maintaining food safety and sustainability in food packaging systems.

Together, these three clusters—material selection and structural optimization (red), functional enhancement through nanotechnology and additives (green), and practical applications in food preservation (blue)—collectively represent the diverse aspects of biopolymer coating research in food packaging. They reflect the ongoing efforts to create sustainable, safe, and high-performance packaging materials that meet the increasing demands for both food quality and environmental responsibility.

### 3.5. Thematic Evolution

**Figure 5:**
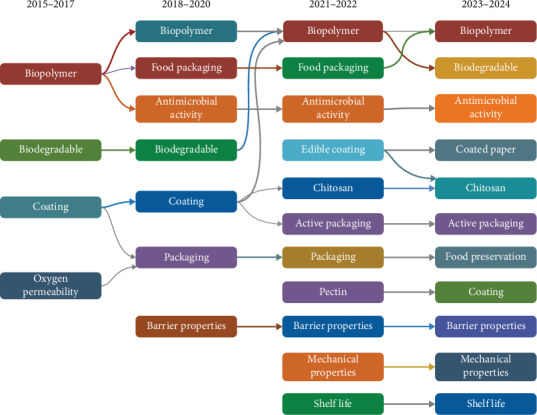
Thematic evolution of biopolymers coating for food packaging from 2015 to 2024 illustrates the progression of key research themes, including biopolymers, coatings, barrier properties, and antimicrobial activity, highlighting evolving trends across time periods.

The thematic evolution of research on biopolymer coatings for food packaging from 2015 to 2024, as illustrated in Figure 5, reveals a clear trajectory from foundational studies to advanced, application-driven innovations. The period from 2015 to 2017 was primarily focused on establishing the basic properties and viability of biopolymer coatings, emphasizing biopolymers, coatings, biodegradability, and oxygen permeability. Studies during this time, like Unalan et al., showed how graphene oxide (GO) could enhance the mechanical and barrier properties of biopolymer films, while Mascheroni et al. explored the potential of CNCs as an alternative to synthetic resins in food packaging [53, 75].

The 2018–2020 period marked a shift toward more application-oriented research, as themes like food packaging, antimicrobial activity, and packaging emerged. This phase highlighted a growing interest in integrating active components into biopolymer coatings for enhanced food preservation and antimicrobial properties. Studies by Shankar and Rhim and Mustapha et al. demonstrated how incorporating essential oils into biopolymer coatings could improve both the mechanical strength and antimicrobial activity of the coatings, extending food shelf life [49, 76]. The development of multilayered packaging using polyhydroxyalkanoates (PHAs) and cellulosic aerogels also marked a significant step in advancing the practical applications of biopolymer coatings for sustainable food packaging.

The 2021–2022 period saw further specialization with a greater focus on specific biopolymers such as CH and pectin. Studies like Al-Moghazy et al. and Jovanović et al. explored CH-based coatings integrated with essential oils to extend shelf life and enhance antimicrobial activity [65, 72]. The rise of terms like edible coatings, active packaging, and the continued importance of biodegradability and mechanical properties pointed to a growing emphasis on functional coatings that offer multiple benefits, such as improving food safety and sustainability. These studies reflect the increasing complexity and specialization of biopolymer coatings, moving beyond basic material characterization to real-world applications in the food industry.

Finally, the 2023–2024 period consolidated these trends, with biopolymer, biodegradable, active packaging, and CH maintaining their central roles. New themes such as coated paper and food preservation indicated a deeper integration of biopolymer coatings into end-use applications. Research like Chettri et al. and Abdalla et al. demonstrated how biopolymer coatings could control moisture and gas exchange, significantly extending the shelf life of fruits and vegetables [57, 77]. The continued focus on shelf life, barrier properties, and mechanical properties highlighted the holistic approach toward developing sustainable, functional, and market-ready packaging solutions.

The evolution from 2015 to 2024 illustrates a shift from exploring the basic viability of biopolymer coatings to developing multifunctional, active packaging solutions that address food preservation, sustainability, and consumer safety. This thematic progression underscores the growing maturity of biopolymer coatings as a sustainable and scalable solution in the food packaging industry.

### 3.6. Research Findings

Research into biopolymer coatings for food packaging has significantly evolved, with a growing focus on sustainable materials such as PLA, CH, cellulose, and PHAs. While these biopolymers offer notable advantages, such as biodegradability and versatility, they also face challenges in terms of mechanical strength and barrier properties compared to conventional plastics [52]. The integration of nanomaterials like ZnONPs and AgNPs has been key in enhancing the performance of these coatings, improving their ability to resist moisture, oxygen, and light. However, these advances raise concerns regarding the environmental impact, particularly the potential for nanoparticle leaching into food products and the environment. As the biopolymer industry progresses, striking a balance between material functionality and environmental safety will be critical for ensuring the long-term viability of these sustainable packaging solutions.

Despite the promising advances in biopolymer coatings, particularly in active packaging and food preservation, significant challenges remain, especially in terms of commercial scalability and cost-effectiveness. The incorporation of bioactive agents such as essential oils into biopolymer matrices has proven effective in extending the shelf life of food products and enhancing food safety [65]. However, transitioning from laboratory settings to mass production remains a substantial hurdle. Furthermore, although biopolymers like PLA and PHA offer biodegradability, their industrial composting requirements and recycling challenges complicate their environmental benefits [29]. The development of a circular economy for biopolymers—where their lifecycle, from production to disposal, is efficiently managed—will be essential in overcoming these challenges. Continued research is needed to reduce production costs and enhance waste management strategies to ensure the economic and environmental sustainability of biopolymer coatings in the food packaging industry.

**Figure 6:**
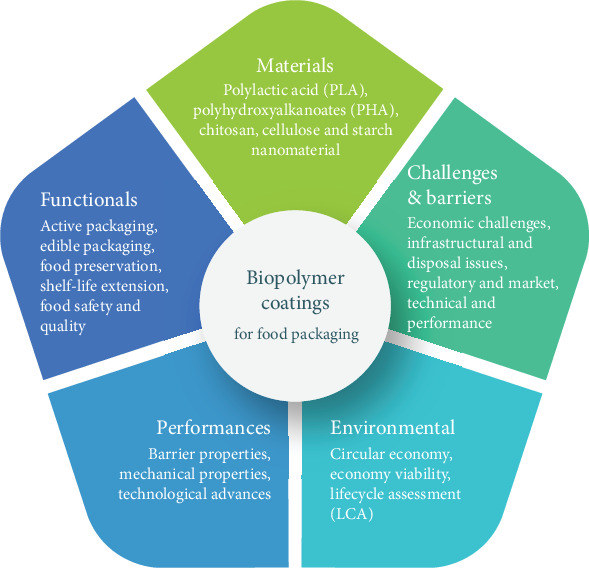
Framework of biopolymer coating studies for food packaging highlights key aspects of biopolymer coatings, including materials, functionalities, performances, environmental impacts, and challenges, emphasizing their role in sustainable food packaging solutions.

This study, illustrated in Figure 6, presents a thorough exploration of the materials, functionalities, environmental impacts, and challenges associated with biopolymer coatings. It examines the potential of biopolymers such as PLA, CH, and cellulose to deliver sustainable packaging solutions that protect food from moisture, oxygen, and other environmental factors while extending shelf life through active and edible packaging technologies. The findings point to the need to improve barrier and mechanical properties and address the economic and infrastructural challenges that limit broader adoption. Adopting a circular economy approach is also essential to strengthening the environmental performance of these materials. Overall, this work provides a clear overview of the current landscape of biopolymer coatings, detailing both recent advancements and the remaining challenges that must be addressed to fully realize their potential in the food packaging industry.

### 3.7. Technological Advancements

#### 3.7.1. Materials Development and Characterization

The development and characterization of biopolymer coatings for food packaging are critical research areas focused on enhancing material properties to meet modern packaging demands. A wide variety of natural and synthetic biopolymers have been studied, each offering distinct advantages as sustainable, functional, and biodegradable alternatives to conventional plastics.

Growing interest in natural biopolymer–based food coatings stems from the urgent need to replace synthetic plastic packaging with eco-friendly, biodegradable solutions. As shown in Table 5, key polysaccharides such as CH, cellulose, starch, alginate, and carrageenan have been extensively investigated for their film-forming ability, biodegradability, and effectiveness as moisture and oxygen barriers. Among these, CH, cellulose, and starch stand out for their abundance, renewable sources, and functional properties, particularly in food preservation and moisture control (CH [41, 78, 79] and cellulose [53, 91, 92]).

CH, derived from shrimp or crab shells, is well known for its antimicrobial properties, making it especially useful for coatings on fresh produce, meat, and seafood. However, CH films alone have drawbacks, including low mechanical strength, limited flexibility, and high moisture sensitivity, which restrict their commercial use [41, 78]. To overcome these limitations, researchers have explored blending CH with alginate, pectin, or CNCs, resulting in films with greater durability, improved oxygen barrier properties, and enhanced overall functionality [53, 91, 92]. Despite these improvements, challenges related to scalability and cost-efficiency continue to hinder the widespread adoption of nanoreinforced CH coatings in commercial food packaging [79, 83].

In contrast, cellulose- and starch-based coatings are widely studied due to their film-forming ability, biodegradability, and cost-effectiveness [64, 88, 89]. However, pure cellulose and starch films suffer from brittleness and high water permeability, which reduce their practical application in high-humidity environments. To overcome these drawbacks, researchers have investigated reinforcement strategies such as CNC incorporation, blending with pectin or alginate, and cross-linking with lipids or proteins to improve barrier properties and mechanical strength [72, 80, 81]. CNCs, derived from acid hydrolysis of plant cellulose, have proven effective in enhancing tensile strength and reducing water sensitivity, making them viable candidates for improving biodegradable food coatings [53, 91]. Similarly, the incorporation of CMC into edible coatings has been shown to improve viscosity, stabilize formulations, and prevent phase separation, making it particularly useful for liquid coatings and food emulsions [42, 52, 85]. These developments suggest that polysaccharide-based coatings could replace petroleum-based films in low-moisture food applications, yet their long-term durability and large-scale processing methods still require further optimization [84, 92].

Beyond polysaccharides, protein- and lipid-based coatings provide additional functional properties, particularly in moisture resistance, oxidative stability, and mechanical flexibility [60, 93, 94]. Zein, casein, whey protein, and soy protein have shown promise in enhancing barrier properties while offering biodegradability and film-forming capabilities [44, 81, 96]. However, proteins alone often lack flexibility and require plasticizers to improve their structural integrity. Meanwhile, lipid-based coatings such as beeswax and carnauba wax effectively reduce moisture loss and extend shelf life but often result in brittle films that require polysaccharide integration for flexibility [44, 100]. The future of food coatings lies in multifunctional hybrid biopolymer systems, where combinations of polysaccharides, proteins, and lipids are optimized to achieve superior mechanical strength, moisture resistance, and food safety performance [84, 97]. While natural biopolymer coatings hold great potential in sustainable food packaging, further research is needed to optimize formulations, improve large-scale processing efficiency, and reduce costs for broader commercial implementation [79, 83].

To address the limitations of natural biopolymer coatings, synthetic biopolymers have emerged as promising alternatives in food packaging applications. These materials offer enhanced mechanical properties, superior moisture and gas barrier performance, and improved stability compared to their natural counterparts. Synthetic biopolymers such as PLA, PHAs, and polybutylene succinate (PBS) provide biodegradable and sustainable solutions while maintaining the functional attributes required for food coatings. The integration of these polymers into food packaging systems allows for greater flexibility, durability, and scalability in commercial applications. Table 5 presents an overview of various synthetic biopolymers utilized for food coatings, detailing their sources, extraction methods, key characteristics, and specific applications in food preservation and protection.

The shift from petroleum-based plastics to biopolymer-based food coatings has accelerated due to environmental concerns and regulatory pressure to promote sustainability. Among the most studied synthetic biopolymers, PLA and PHAs have gained prominence due to their biodegradability and potential to replace conventional plastic films. PLA and PHAs are produced through distinct methods that set them apart from other biodegradable biopolymers. PLA is synthesized via the polymerization of lactic acid, typically derived from cornstarch, while PHAs are produced by microbial fermentation of organic substrates. This reliance on specific renewable resources and microbial processes categorizes them as synthetic biopolymers [101]. As outlined in Table 6, these biopolymers offer advantages such as renewable sourcing, mechanical strength, and suitability for direct food contact. However, PLA suffers from brittleness and limited flexibility, whereas PHA has high production costs, making its large-scale adoption challenging. To improve their mechanical and barrier properties, researchers have developed hybrid approaches, blending these materials with PBS, polycaprolactone (PCL), and polybutylene adipate-co-terephthalate (PBAT) to enhance flexibility, durability, and processing efficiency.

PLA is one of the most widely used biodegradable thermoplastic polymers, produced through fermentation of sugars from starch (corn and sugarcane) followed by polymerization into polylactide [58]. Its strength, oil resistance, and compostability make it an attractive option for food coatings in bakery products and confectionery. However, PLA's brittleness and low gas barrier properties limit its application in flexible packaging. Blending PLA with more flexible biopolymers, such as PBS and PBAT, enhances its elasticity while maintaining biodegradability and food safety compliance [55]. Additionally, nanomaterial reinforcements using CNCs or clay nanoparticles have been investigated to improve their moisture resistance and mechanical performance.

On the other hand, PHAs, produced via microbial fermentation, offer superior flexibility and resistance to water and oils, making them ideal for food packaging applications requiring higher durability [103]. Unlike PLA, PHA is biodegradable in both soil and marine environments, making it a strong candidate for sustainable packaging solutions. However, PHA's high production cost and variability in polymer properties pose significant commercialization challenges. Recent studies have explored copolymerization with polyhydroxybutyrate (PHB) and polyhydroxyvalerate (PHV) to enhance mechanical strength and processability. Furthermore, PHA-PBS blends have demonstrated improved flexibility and cost efficiency, expanding their potential applications in biodegradable food wrapping.

Beyond PLA and PHA, PBS and PCL have gained attention for their flexibility and thermal stability. PBS, synthesized from succinic acid and butanediol, is biodegradable, fat-resistant, and thermally stable, making it suitable for protective coatings on dry foods and bakery products [104, 105]. Similarly, PCL, a highly flexible and chemically stable polymer, has been incorporated into edible coatings for processed foods and as a biopolymer base for packaging applications [106]. Despite these advantages, both PBS and PCL have relatively slow degradation rates, limiting their use in short-term food packaging. Blending with PLA or PBAT enhances their biodegradability while maintaining high mechanical flexibility.

Finally, PBAT and polyvinyl alcohol (PVA/PVOH) provide unique functionalities in biodegradable coatings and edible packaging applications. PBAT's rubber-like flexibility and oil resistance make it an ideal substitute for PE in food wraps [105]. However, its poor oxygen barrier properties require blending with PLA or PHA to improve protection against oxidation and moisture loss. Meanwhile, PVA, a water-soluble biodegradable polymer, is widely used in soluble food packaging, edible films, and food coatings [43, 110]. Despite its advantages, PVA's solubility can be a drawback in humid environments, necessitating modifications such as cross-linking with hydrophobic polymers. As advancements in biopolymer technology and material engineering continue, hybrid polymer formulations and nanotechnology integration will be crucial in optimizing performance, cost-effectiveness, and large-scale industrial adoption of synthetic biopolymer–based food coatings.

#### 3.7.2. Integration of Nanomaterials in Biopolymer Coatings

Incorporating nanomaterials into biopolymer coatings is a key research focus aimed at improving mechanical strength, thermal stability, and barrier performance. These nanomaterials act as reinforcements, enabling the use of thinner coatings while maintaining the necessary protection, flexibility, and durability required for food packaging applications. As shown in Table 7, the most commonly used nanomaterials include nanocellulose, nanoclays, metal-based nanoparticles, carbon-based nanomaterials, and nanolipids—each contributing distinct benefits to enhance the overall performance of biopolymer coatings.

Nanocellulose (CNCs and CNFs): Derived from plant cellulose, CNCs and cellulose nanofibers (CNFs) significantly improve mechanical strength, flexibility, and oxygen barrier properties in biopolymer films. CNCs, due to their high crystallinity, provide rigid reinforcement, whereas CNFs offer flexibility. These nanocellulose materials are widely blended with PLA, starch, and CH coatings to improve shelf life and sustainability in food packaging [53, 92, 111].

Nanoclays: Layered silicate–based nanomaterials such as montmorillonite (MMT), kaolinite, and halloysite nanotubes (HNTs) enhance gas barrier properties, mechanical strength, and thermal resistance. When incorporated into starch, PVA, or PLA coatings, nanoclays create tortuous pathways, which are intricate, winding routes that oxygen and moisture must travel through, increasing resistance to their permeation. This mechanism improves food preservation and freshness by slowing down the rate at which gases and moisture interact with the packaged food [112–114, 118].

Metal-based nanoparticles (AgNPs, ZnO, and titanium dioxide [TiO_2_]): AgNPs, ZnO, and TiO_2_ are antimicrobial nanomaterials integrated into CH, PHA, and PLA coatings to inhibit bacterial and fungal growth. These nanoparticles extend shelf life by preventing food contamination while also providing UV resistance for better food storage conditions [52, 69, 112].

Graphene-based nanomaterials, particularly GO, are increasingly integrated into biopolymer films to enhance their mechanical strength, conductivity, and oxygen barrier properties, with pullulan/GO nanocomposites offering exceptional oxygen barrier performance, improved mechanical flexibility, and optical clarity, thereby significantly boosting the effectiveness of high-performance food packaging materials [75]. Similarly, GO-reinforced biopolymer coatings, such as starch- or PVA-based films, offer superior moisture resistance, improved structural integrity, and enhanced barrier performance, making them highly effective in extending food shelf life. Moreover, the incorporation of graphene nanomaterials helps reduce water vapor transmission while maintaining flexibility and transparency, further boosting their potential for sustainable food packaging solutions [104, 117].

#### 3.7.3. Functionality and Performance Enhancement

The functionality and performance of biopolymer coatings in food packaging are crucial for their viability as alternatives to conventional plastics. Research in this area focuses on enhancing the barrier properties and mechanical strength of materials and incorporating active packaging features such as antimicrobial and antioxidant activities. Enhancing these properties is essential not only for protecting food products but also for extending their shelf life and maintaining their quality. For example, the inclusion of nanoclay particles in biopolymer matrices has significantly improved the barrier properties by reducing the permeability of gases and moisture [2, 119].

Mechanical strength is another critical area where biopolymer coatings must be improved to compete with conventional plastics. Techniques such as the incorporation of nanomaterials like CNCs and natural fibers have been shown to significantly enhance the tensile strength and flexibility of biopolymer films, making them more suitable for a wider range of packaging applications [41, 120]. Additionally, blending biopolymers with other biodegradable polymers like PHAs creates biocomposite materials with mechanical properties that closely approximate those of conventional plastics [120]. Biocomposites have garnered significant interest from scientists and engineers due to their abundant availability, low carbon footprint, and biodegradability [121].

Active packaging systems incorporating antimicrobial and antioxidant agents are another area of focus in biopolymer research. These systems not only protect food from external factors but also actively preserve food quality by preventing microbial growth and oxidation. For instance, CH-based coatings have proven effective in extending the shelf life of perishable foods like strawberries by inhibiting microbial contamination [51, 62]. Similarly, the integration of antioxidant compounds such as tocopherols and ascorbic acid into biopolymer coatings helps prevent food spoilage, thereby reducing food waste [22, 80].

Edible packaging is an innovative application of biopolymer coatings that not only serves as a protective barrier but can also be safely consumed along with food to reduce environmental waste. Edible coatings made from natural biopolymers like polysaccharides, proteins, and lipids provide effective barriers against moisture, oxygen, and microbial contamination, making them particularly beneficial for fresh produce and ready-to-eat foods [80, 81]. The incorporation of active ingredients such as antimicrobial agents and antioxidants further enhances the protective functions of edible packaging, extending the shelf life of food products while maintaining safety and quality [22, 80].

Recent technological advancements have significantly enhanced the performance of biopolymer coatings, making them more viable as sustainable alternatives to conventional plastic packaging. Innovations such as the incorporation of nanomaterials, the development of active packaging systems, and the use of advanced processing techniques like electrospinning have broadened the application potential of biopolymer coatings [15, 112]. These advances not only improve the mechanical strength, barrier properties, and thermal stability of biopolymers but also open new possibilities for their application in more demanding packaging scenarios, thereby pushing the boundaries of sustainable packaging solutions [52, 53].

### 3.8. Environmental Impact and Lifecycle Analysis

Biopolymer coatings offer substantial environmental benefits over conventional plastics, primarily because of their origins in renewable resources. Polymers such as PLA and PHAs are derived from agricultural feedstocks like corn and sugarcane, which absorb carbon dioxide (CO_2_) from the atmosphere during their growth. This natural carbon sequestration significantly reduces the overall carbon footprint of biopolymers compared to petroleum-based plastics. As a result, the production and use of biopolymers contribute to reducing greenhouse gas emissions, which aligns with global sustainability goals [7, 13].

However, the environmental advantages of biopolymer coatings are closely linked to the conditions under which they are disposed of. Biopolymers like PLA and PHA are designed to biodegrade under specific conditions, which are typically found in industrial composting facilities. These facilities maintain controlled levels of temperature, humidity, and microbial activity, which are essential for the efficient breakdown of biopolymers into nontoxic components that can be reintegrated into natural ecosystems. In regions where industrial composting infrastructure is well developed, the environmental benefits of biopolymers are fully realized because these materials can be effectively composted, reducing waste and minimizing environmental impact [2, 22, 122].

Conversely, in areas where industrial composting facilities are underdeveloped or nonexistent, the environmental benefits of biopolymers may be significantly diminished. Without access to proper composting conditions, biopolymers may not degrade as intended, leading to environmental challenges similar to those of conventional plastics. In such cases, biopolymers may persist in the environment, contributing to pollution and undermining their sustainable potential [13–15]. This highlights the critical need for expanding composting infrastructures and improving waste management systems to fully capitalize on the environmental advantages of biopolymer coatings.

Furthermore, comprehensive lifecycle assessments (LCAs) are crucial for evaluating the sustainability of biopolymer coatings. LCAs consider various factors, such as energy consumption, water use, and greenhouse gas emissions, throughout the lifecycle of biopolymers, from production to disposal. Although biopolymers generally perform well at reducing carbon emissions, their overall environmental impact depends heavily on the availability of appropriate disposal facilities. Therefore, to maximize the sustainability of biopolymer coatings, it is essential to address infrastructural challenges and promote the development of robust industrial composting systems worldwide [7, 17].

### 3.9. Challenges and Barriers to Adoption

Biopolymer coatings offer significant environmental benefits, such as reduced carbon footprints and biodegradability, but these advantages rely heavily on adequate composting infrastructure. Without proper facilities, biopolymers like PLA and PHA may not degrade as intended, leading to environmental persistence similar to conventional plastics [7, 13]. Many regions lack the industrial composting infrastructure necessary for biopolymers to fully break down, which undermines their sustainability potential [17, 122].

To overcome this, expanding industrial composting facilities is critical. Governments and industries should prioritize investment in composting infrastructure, potentially through public–private partnerships that offer incentives for private sector involvement in building such facilities [2, 13]. This development would ensure biopolymer coatings can degrade effectively, maximizing their environmental benefits.

Increased consumer education is also essential, as many consumers struggle to distinguish between biodegradable and nonbiodegradable products. Public awareness campaigns and clearer labeling can promote proper disposal practices, further enhancing the environmental impact of biopolymer products [7, 17].

Additionally, research should focus on improving the biodegradability of biopolymers in various environments, reducing their reliance on industrial composting. This includes developing materials that degrade in home composting or natural settings [14, 15]. Regulatory support, including policies mandating biodegradable packaging and standardized certifications for compostable materials, will also be crucial in promoting the widespread adoption of biopolymer coatings [13].

### 3.10. Future Direction and Research Gaps

#### 3.10.1. Identified Research Gaps

The bibliometric analysis reveals several gaps in biopolymer coating research that must be addressed to advance the field. Scalability remains a key gap, with limited research on the mechanical properties, durability, and cost-effectiveness of biopolymers at a large scale. This gap hinders their widespread adoption in food packaging. Additionally, current production processes for biopolymers are energy-intensive and costly, which limits their competitiveness with conventional plastics. Research into more efficient production methods, such as integrating renewable energy or advancing biorefining technologies, is needed to reduce environmental impact and costs.

Another gap involves understanding the long-term environmental impact of biopolymer coatings in diverse disposal environments. Although biodegradability is a major advantage, the conditions required for effective degradation are not always present in real-world scenarios. Further research is necessary to explore how factors like temperature, humidity, and microbial presence affect biodegradation and the overall environmental footprint of biopolymers.

#### 3.10.2. Suggestion for Future Research

To address the identified gaps, future research should focus on several key areas. Scalability must be a primary concern, with studies examining ways to produce biopolymer coatings at an industrial scale while preserving their desirable properties. This includes exploring new manufacturing methods, such as continuous processing and advanced material formulations, to improve production efficiency and reduce costs.

Optimizing production methods is equally important in advancing biopolymer research. Future studies should explore ways to streamline the production process, potentially by incorporating more sustainable energy sources or developing new catalysts and enzymes that enable polymerization under milder conditions. These advancements could substantially reduce the energy requirements and environmental impact of biopolymer production.

Additionally, comprehensive LCAs are needed to evaluate the environmental impact of biopolymer coatings from production to disposal. These studies should consider different disposal environments and assess the biodegradability and ecological effects of these materials over time. Such assessments will provide a deeper understanding of the performance of biopolymers beyond controlled laboratory conditions, helping to inform better design practices and policy decisions.

## 4. Conclusion

The field of biopolymer coatings for food packaging has emerged as a rapidly expanding area of research, catalyzed by escalating global environmental concerns and the imperative to transition toward sustainable packaging systems. This study reveals significant progress in the development and application of biodegradable polymers—particularly PLA, PHAs, and CH—which are increasingly recognized as viable alternatives to conventional petroleum-based plastics. These biopolymers exhibit favorable barrier properties against moisture, oxygen, and microbial infiltration while also aligning with circular economy principles by reducing dependency on nonrenewable resources. Recent innovations have further enhanced the performance of these materials through the incorporation of nanomaterials and bio-based functional additives, which improve mechanical integrity, thermal stability, and active functionalities, thereby broadening their potential for commercial-scale applications. Despite these advancements, critical barriers persist, including elevated production costs, underdeveloped industrial composting infrastructure, and insufficient public awareness regarding end-of-life management. The bibliometric analysis presented in this study illustrates a marked increase in global scholarly output and citation frequency related to biopolymer coatings between 2015 and 2024. This trend reflects both the intensifying scientific interest and the strategic relevance of this research area in addressing environmental sustainability. Moreover, international collaboration—particularly among leading research-producing countries such as Brazil and China—demonstrates a shared global commitment to innovating sustainable packaging technologies. As the field evolves, there is a discernible pivot toward application-oriented studies focused on optimizing functionality, processability, and industrial scalability. The convergence of materials science innovation with policy and infrastructure development underscores the pivotal role that biopolymer coatings are expected to play in the transition toward environmentally responsible food packaging solutions.

## Figures and Tables

**Table 1:** Top 10 most prominent journals.

**Rank**	**Journal**	**Papers**	**Citations**	**h** **-index**	**CiteScore 2024**	**Most cited paper**
1	*International Journal of Biological Macromolecules*	31	1275	19	10.3 (Q1)	[41]
2	*Polymers*	18	370	10	9.7 (Q1)	[42]
3	*Journal of Applied Polymer Science*	10	273	8	5.8 (Q2)	[43]
4	*Food Chemistry*	9	576	9	18.3 (Q1)	[44]
5	*Food Packaging and Shelf Life*	8	341	8	16.2 (Q1)	[45]
6	*Coating*	7	191	4	5.4 (Q2)	[46]
7	*Progress in Organic Coatings*	7	175	6	11.8 (Q1)	[47]
8	*Food Hydrocolloids*	5	233	5	21.7 (Q1)	[48]
9	*Carbohydrate Polymers*	5	203	5	24.0 (Q1)	[49]
10	*Journal of the Science of Food and Agriculture*	5	144	5	7.9 (Q1)	[50]

**Table 2:** Top 10 countries in biopolymer research based on the number of publications.

**Country**	**Papers**	**Citations**	**Most cited paper**
Brazil	29	1265	Espitia et al. [51]
China	28	833	Guo et al. [52]
Italy	27	985	Mascheroni et al. [53]
India	27	579	Naqash et al. [54]
Spain	23	139	Benito-Gonzáles et al. [55]
France	13	335	Fotie et al. [56]
United States	12	292	Abdalla et al. [57]
Germany	10	241	Habel et al. [58]
Thailand	9	330	Adhikari et al. [59]
Iran	8	251	Hosseini et al. [60]

**Table 3:** Top 10 influential papers and their research findings.

**Paper**	**Citation**	**Research finding**
Mascheroni et al. [53]	170	CNC coatings, produced using ammonium persulfate, improved oxygen barrier properties and transparency compared to sulfuric acid–treated CNCs, offering potential for eco-friendly food packaging.
Homez-Jara et al. [41]	164	Lower drying temperatures improved moisture content and solubility in chitosan coatings, while higher temperatures and chitosan concentration enhanced tensile strength and swelling power for food packaging.
Espitia et al. [51]	162	Nanoemulsions enhance biopolymer coatings in food packaging by improving stability, bioavailability, and antimicrobial properties, enabling the incorporation of bioactive compounds for sustainable solutions.
Cardoso et al. [61]	149	Chitosan–gelatin coatings effectively reduced weight loss and lipid oxidation in beef, with higher gelatin concentrations being more effective.
Pavinatto et al. [62]	140	Coating with chitosan-based films provided antibacterial protection to strawberries, maintaining their quality.
Kopacic et al. [63]	113	Alginate and chitosan coatings on paperboard improved grease resistance, reduced water vapor transmission, and enhanced barrier properties, making them suitable for sustainable food packaging.
Shankar and Rhim [49]	99	Biopolymer-coated paper with alginate, carboxymethyl cellulose, and carrageenan enhanced water, oil, and vapor resistance while offering antibacterial properties, making it ideal for sustainable food packaging.
Dhumal et al. [64]	97	Biphasic sago starch–guar gum coatings with essential oils exhibited antimicrobial properties, improved water barrier performance, and potential for use in active food packaging.
Klunklin et al. [42]	87	*Asparagus* stalk–derived carboxymethyl cellulose (CMCas) films, synthesized with varying NaOH concentrations, showed excellent mechanical properties and potential applications in coatings and food packaging.
Al-Moghazy et al. [65]	84	Chitosan-based coatings with thyme essential oil encapsulated in liposomes extended Karish cheese's shelf life, maintaining microbial safety for up to 4 weeks with minimal effects on quality.

**Table 4:** Thematic clusters from co-occurrence analysis of keywords in biopolymer coating research.

**Cluster**	**Themes**	**Main keywords**
Red	Biopolymers for sustainable food packaging	Biopolymers, Food Packaging, Coatings, Packaging, Cellulose, Starch, Alginates, Polysaccharides, Biodegradable Polymers, Biodegradable, Active Packaging, Antimicrobial, Barrier properties, Mechanical Properties, Mechanical permeability
Green	Functional properties and material characterization	Chemistry, Tensile Strength, Scanning Electron Microscopy, Fourier Transform Infrared Spectroscopy, Water Vapor, Moisture, Permeability, Hydrophobicity, Surface Properties, Anti-microbial Activity, *Escherichia coli*, Nanoparticles, Nanocomposites, Moisture, X-ray Diffraction, Antiinfective Agents
Blue	Food preservation, safety, and shelf life	Chitosan, Fruits, Food Preservation, Edible Coatings, Edible Packaging, Shelf Life, Essential Oils, Glycerol, Hydroxypropyl Methylcellulose, Shelf Life, Antioxidants, Antioxidant Activities, Food Safety, Pectin

**Table 5:** Natural biopolymers for food coatings.

**Category**	**Biopolymer**	**Source and extraction**	**Characteristics**	**Application in food coatings**	**Ref.**
Polysaccharide	Chitosan	Extracted from shrimp or crab shells	Antimicrobial, film-forming, biodegradable	Used in antimicrobial coatings for fresh produce, meat, and seafood	[41, 78, 79]
	Pectin	Derived from fruit peels (apple, citrus)	Gel-forming, thickener, biodegradable	Used in coatings for fruits, dairy products, and jellies	[72, 80, 81]
	Pullulan	Produced by the fermentation of starch using *Aureobasidium pullulans*	Highly transparent, excellent oxygen barrier, flexible, and edible	Used in edible films, food wraps, and biodegradable packaging for snacks and candies	[82]
	Alginate	Extracted from brown algae	Gel-forming, moisture retention, oxygen barrier	Used in coatings for fresh produce and seafood	[63, 83, 84]
	Carrageenan	Extracted from red seaweed	Gelling, thickening, and stabilizing properties, forms strong hydrocolloid	Used in dairy coatings, meat preservation, and edible coatings for processed foods	[49, 83]
	Carboxymethyl cellulose (CMC)	Derived from cellulose via chemical modification	Improves viscosity, stabilizes film coatings	Used in stabilizing edible coatings for food and beverages	[42, 52, 85]
	Arabic gum	Extracted from *Acacia* tree exudates	Emulsifier, film-forming, biodegradable	Used in candy coatings, beverage stabilization	[86, 87]
	Starch	Extracted from corn, cassava, potato, or sago	Biodegradable, good film-forming properties	Used in edible coatings for fruits, vegetables, and bakery products	[64, 88–90]
	Cellulose nanocrystals (CNCs)	Derived from plant cellulose via acid hydrolysis	Highly crystalline, strong, improves mechanical properties, enhances barrier properties	Used as reinforcement in edible films, enhances oxygen and moisture barrier in biopolymer coatings	[53, 91, 92]

Protein	Zein	Derived from corn protein	Transparent, water-resistant, high oxygen barrier	Used in coatings for nuts, snacks, and confectionery	[60, 93, 94]
	Casein	Extracted from milk protein	Moisture barrier, biodegradable	Used in coatings to reduce moisture transfer in cheese and processed foods	[95]
	Whey protein	Byproduct of cheese production	Improves oil and water resistance, film-forming	Used in edible films for meat, dairy, and snack coatings	[81, 96]
	Soy protein	Extracted from soybean	Strengthens film, prevents oxidation	Used in coatings for plant-based foods and fruits	[97, 98]

Lipid	Beeswax	Extracted from honeybee combs	Hydrophobic, glossy protective layer	Used in coatings for fruits (apples, citrus) to prevent dehydration	[44, 99]
	Carnauba wax	Extracted from Carnauba palm leaves	Water-resistant, biodegradable, glossy	Used in fruit coatings (apples, citrus) and candy coatings	[100]

**Table 6:** Synthetic biopolymers for food coatings.

**Biopolymer name**	**Source & extraction**	**Characteristics**	**Application in food coating**	**Ref.**
Polylactic acid (PLA)	Produced through fermentation of sugars from starch (corn, sugarcane) by lactic acid bacteria, followed by polymerization into polylactide	Biodegradable, transparent, strong, oil-resistant, thermoplastic properties	Used for protective food packaging and edible films in confectionery and bakery products	[55, 58, 102]
Polyhydroxyalkanoates (PHAs)	Produced by microorganisms such as *Cupriavidus necator* through fermentation of sugars or vegetable oils, then purified via extraction	Biodegradable, flexible, resistant to oil and water, high mechanical strength	Used as a plastic substitute in edible food packaging and food wrapping	[103]
Polybutylene succinate (PBS)	Synthesized from succinic acid and 1,4-butanediol through esterification polymerization	Biodegradable, flexible, thermally stable, resistant to fats	Used for protective coatings on dry foods, bakery products, and flexible packaging layers	[104, 105]
Polycaprolactone (PCL)	Synthesized via polymerization of cyclic ester caprolactone	Biodegradable, highly flexible, chemically stable	Used in edible coatings for processed foods and biopolymer-based packaging	[106–108]
Polybutylene adipate-co-terephthalate (PBAT)	Produced through the copolymerization of butylene adipate and butylene terephthalate	Biodegradable, flexible, oil-resistant, with mechanical properties similar to conventional plastics	Used in biodegradable edible films and food wrapping	[105, 109]
Polyvinyl alcohol (PVA/PVOH)	Produced through the polymerization of vinyl acetate, followed by hydrolysis to polyvinyl alcohol	Water-resistant, flexible, biodegradable, good film-forming properties	Used in food coatings and soluble food packaging	[43, 110]

**Table 7:** Types of nanomaterials in biopolymer coatings.

**Nanomaterial**	**Category**	**Function in biopolymer coatings**	**Examples of biopolymers used**	**Key advantages**	**Ref.**
Cellulose nanocrystals (CNCs)	Polysaccharide-based	Enhances mechanical strength, oxygen barrier, and flexibility	PLA, starch, chitosan	Improves film durability and reduces permeability	[53, 92]
Cellulose nanofibers (CNFs)	Polysaccharide-based	Provides flexibility, biodegradability, and water resistance	Starch, alginate, chitosan	Reduces moisture sensitivity of coatings	[111]
Montmorillonite (MMT)	Nanoclay	Improves gas barrier properties and thermal stability	PVA, PLA, starch	Delays oxygen and moisture transmission	[112, 113]
Halloysite nanotubes (HNTs)	Nanoclay	Enhances strength, water retention, and adsorption properties	PLA, starch, pectin	Improves coating toughness	[95, 114]
Silver nanoparticles (AgNPs)	Metal-based	Provides antimicrobial activity, extends shelf life	Chitosan, PHA, starch	Inhibits bacterial growth, enhances food safety	[69, 112]
Zinc oxide (ZnO)	Metal-based	Offers UV protection, antibacterial properties	PLA, chitosan, pectin	Reduces food oxidation and microbial spoilage	[52, 112]
Titanium dioxide (TiO_2_)	Metal-based	Enhances UV resistance and antimicrobial effect	PLA, starch, alginate	Preserves food quality and stability	[112, 115, 116]
Graphene oxide (GO)	Carbon-based	Improves mechanical durability, conductivity, and oxygen barrier	Starch, PVA, chitosan	Provides stronger film flexibility	[104, 117]

## Data Availability

The data used to support the findings of this study are available from the corresponding author upon request.
